# The role of rarity as a surrogate of marine fish species representation

**DOI:** 10.7717/peerj.8373

**Published:** 2020-02-10

**Authors:** Fabio Albuquerque, Yaiyr Astudillo-Scalia

**Affiliations:** Science and Mathematics Faculty, College of Integrative Sciences and Arts, Arizona State University, Mesa, AZ, United States of America

**Keywords:** Rarity, Surrogates of biodiversity, Rarity weighted richness, Index of summed rarity, Index of relative rarity, Marine fishes, Alpha diversity

## Abstract

Because the distribution of most of the species is poorly known, conservationists use surrogates to help maximize the representation level of all species. Historically, species richness has been used to calculate the importance of priority areas for conservation, but several studies revealed sites with high species richness often fail to determine the smallest number of sites that will protect the highest number of species. Rarity, however, has played a prominent role in safeguarding planning units. While the performance of rarity has been previously assessed in terrestrial systems, we tested the hypothesis that rarity of a site can be used as a measure of the importance of a site to a conservation network in marine ecosystems. We used the presence data (at a 1-degree resolution) to calculate five rarity indices of fish diversity at a global extent and compared the results to those obtained by using species richness and site complementarity. Our objectives were to: (1) determine if rarity indices can be used as surrogates of fish biodiversity by representing the highest number of species in the smallest number of sites; and (2) determine if the effectiveness of these indices to represent fish biodiversity is impacted by the metric used to define rarity. Results indicate that rarity could be an effective surrogate for marine fishes, as most results showed a mean of 100% effectiveness. In the context of marine biodiversity conservation, results show that rarity indices could be considered affordable and feasible surrogates of species representation, with the most significant benefit to those areas of the world that are in most need to access alternative tools. Results also open a new area of collaboration between biogeography and marine conservation biology since planners can use biogeographical patterns of rarity to enhance the performance of the current protected area network.

## Introduction

Because the geographical distribution of most marine species is poorly understood (the Wallacean shortfall), biogeographers, conservation planners and stakeholders often use surrogates (i.e., a measurement that can be used as a substitute for biodiversity in a given area) to prioritize sites (Smith, 2005; Rodrigues & Brooks, 2007). Environmental categories (e.g., environmental diversity) and taxonomic data (e.g., plant diversity) are widely used as biodiversity surrogates to predict the overall diversity of a particular region and to identify priority areas for conservation (see e.g.,  Myers et al., 2000; Mellin et al., 2011; Albuquerque & Beier, 2015a; Sutcliffe et al., 2015; Beier & Albuquerque, 2016). The main idea behind the use and the utility of surrogates in monitoring programs is that substitutes for biodiversity will help prioritize additional or unknown species and should maximize the representation level of all species (Rodrigues & Brooks, 2007; Sato et al., 2019).

Even if surrogacy is an accepted approach in some conservation action contexts, the effectiveness of surrogates has been assessed by several studies (e.g.,  Rodrigues & Brooks, 2007; Beier et al., 2015). This is because there is not a perfect surrogate that will work in all conservation planning scenarios, and testing of different surrogates on a case-by-case basis is required to determine the most appropriate one (Rodrigues & Brooks, 2007; Mellin et al., 2011). One example is species richness (the number of species at a site). For about 30 years, conservation biologists have used the patterns of species richness at global, continental, and different extents to draw implications for conservation of biodiversity (Ladle & Whittaker, 2011). Richness is the most used descriptor of a site’s biodiversity and has been widely used for restoring perturbed communities (Ladle & Whittaker, 2011; Magurran & McGill, 2011; Ramírez et al., 2017). But species richness has limited relevance to conservation prioritization (Orme et al., 2005). Sites with a high number of species frequently encompass overlapping communities, and as a consequence, species-rich sites fail to represent the majority of species in a small number of sites (Kirkpatrick, 1983; Csuti, 1997; Albuquerque & Beier, 2015a; Albuquerque & Beier, 2015b, and papers cited therein). Consequently, for the last two decades, conservation planners have used complementarity, a measure of sites’ importance that ensure the inclusion of new attributes into an existing reserve network (e.g., Williams et al., 1996; Justus & Sarkar, 2002; Moilanen, Possingham & Polasky, 2009). Several algorithms, such as integer programming (Haight & Snyder, 2009) or heuristic reserve-selection algorithms (Zonation, Moilanen et al., 2014), can be used to estimate complementarity based on conservation scores.

Among the conceptual frameworks that use surrogates to ensure that biodiversity receives some level of conservation actions, the rarity of a site (hereafter rarity) has played a prominent role in protecting planning units (Lawler et al., 2003; Albuquerque & Beier, 2015c; Astudillo-Scalia & Albuquerque, 2019). Rarity can be measured as the proportion, or the sum, of species with the lowest geographical range or abundance in a community (Gaston, 1994; Williams et al., 1996). In addition, rarity gives a higher conservation value to sites with a higher number of limited-range species and has been used in the past in conservation studies of imperiled species (Williams et al., 1996; Stein, Kutner & Adams, 2000; Lawler et al., 2003; Soberón & Ceballos, 2011; Villalobos et al., 2013; Guerin & Lowe, 2015). A previous study of coral reef fishes at the global scale found low agreement between hotspots of rarity and richness and suggested that rarity should be considered for developing conservation strategies of marine fishes (Grenie et al., 2018). Further understanding of rarity as a surrogate of species representation will enhance our knowledge for dealing with biodiversity loss (Ceballos et al., 2015).

One example of a rarity metric is rarity weighted richness (RWR, see Williams et al., 1996 for details). RWR identifies sites with a high-concentration of limited-range species (Stein, Kutner & Adams, 2000). For eleven datasets, including 6,400 animal and plant species mapped across 99,600 sites in different parts of the world, Albuquerque & Beier (2015c) reported that sites accumulated in order of RWR represented species almost as effectively as sets of sites identified by complementarity. They suggested that its simplicity made RWR a simple and reliable alternative to integer programming and heuristic algorithms for representing all species in the smallest number of sites.

Leroy et al. (2012); Leroy, Canard & Ysnel (2013) developed the index of summed rarity (ISR), and the index of relative rarity (IRR). In the former, rarity is defined by the sum of weights of all species, whereas IRR uses a percentage of the maximum occurrence to identify a cut-off threshold to calculate the proportion of species that are rare (Leroy et al., 2012; Leroy, Canard & Ysnel, 2013). Rare species’ weights are expected to increase exponentially when the occurrence of species decreases below the threshold (Leroy et al., 2012). Leroy, Canard & Ysnel (2013) suggested that the IRR approach is a more accurate description of the rarity patterns of assemblages. Astudillo-Scalia & Albuquerque (2019) used multiple datasets to evaluate the performance of IRR and ISR for species representation in conservation planning, and they showed that rarity is a highly efficient surrogate of plants and vertebrates in temperate and tropical terrestrial environments (Albuquerque & Beier, 2015c; Albuquerque & Beier, 2016; Astudillo-Scalia & Albuquerque, 2019). Although previous studies have shown that rarity is a reliable alternative to complex algorithms for representing most species in a small number of sites, tests of these ideas have focused on terrestrial species. Therefore, further studies are required to verify the ability of rarity to maximize the representation level of species in marine ecosystems.

Herein, we tested the hypothesis that rarity can be used as a measure of the importance of a site to a conservation network in marine ecosystems (Albuquerque & Beier, 2015c; Astudillo-Scalia & Albuquerque, 2019). Our main goals were to: (1) determine whether rarity indices can be used as surrogates of fish biodiversity; and (2) investigate if the metric used to define rarity impacts the effectiveness of rarity indices to represent fish biodiversity. If effective, our results can improve our understanding and the use of rarity as a surrogate of marine biodiversity and provide better guidelines for the use of surrogates in applied conservation planning.

## Materials and Methods

### Data

We obtained range maps from the IUCN Red List Spatial Database (IUCN, 2019a; IUCN, 2019b) for 1,104 Chondrichthyes (shark and rays) and 2,558 Osteichthyes. These datasets provide geographical range maps for comprehensively assessed taxonomic groups (e.g., angelfishes, damselfishes, and groupers), and they are produced by combining data from all known expert-verified distribution points (IUCN, 2019a; IUCN, 2019b). Most of the species were assessed as least concern (2,453, 65%) and data deficient species (760, 20%). Datasets also included 245 vulnerable species (7%), 164 near threatened (4%), 88 endangered (2%), and 59 critically endangered species (2%). We used the R package *letsR* (Vilela & Villalobos, 2015) to obtain a map of presence and absence records for each 1° grid cell.

### Defining rarity of a site

We used the presence data, obtained from IUCN range maps, to calculate five rarity indices (rarity of sites). Following Williams et al. (1996), we used the RWR algorithm to estimate the rarity of sites. Specifically, we calculated the inverse of the number of sites in which each species occurs, and we summed the rarity scores of all species present at that site (Williams et al., 1996; Stein, Kutner & Adams, 2000; Beier & Albuquerque, 2016). RWR gives high scores to species with the most limited ranges (e.g., a species present in one site only would have a score of 1).

We followed Leroy et al. (2012) and Leroy, Canard & Ysnel (2013) to calculate the index of relative rarity (IRR) and index of summed rarity (ISR). To do so, we first selected the first quartile of species occurrences as the cut-off point below which a species is considered rare (Gaston, 1994; Leroy, Canard & Ysnel, 2013). Then, we used the *rWeights* function in R (R Core Team, 2019; Leroy, 2016) and the weighted functions *W* and *invQ* (Leroy et al., 2012) to calculate the rarity weight for a single species. In the *W* function, the weights of species with rarity scores below the cut-off point are expected to increase exponentially. Otherwise, weights tend to be zero (Leroy, Canard & Ysnel, 2013). The inv*Q* function is similar to RWR and it is defined by the inverse of the occurrence.

We used the species’ rarity weights produced by *W* and inv*Q*, and the *Irr* and *Isr* functions in R (R Core Team, 2019; Leroy, 2016) to calculate four indices: IRR_W, IRR_InvQ, ISR_W, and ISR_InvQ.

### Evaluating the effectiveness of rarity as a surrogate in prioritization

We used species accumulation curves (SAC) and the species accumulation index (SAI—Rodrigues & Brooks, 2007), to evaluate if rarity indices (RWR, IRR_W, IRR_InvQ, ISR_W, and ISR_InvQ) are effective at representing marine fish biodiversity. For each index, we built SAC by assembling sites with the highest rarity scores and subsequently added sites with the next most top rarity scores after each iteration. We then calculated the number of species represented in at least one cell (*S*). We used the basic core-area Zonation approach (CAZ, Moilanen et al., 2014) to calculate the highest number of species represented in that same number of sites (*O*). The CAZ approach is a heuristic algorithm, which conserves areas that maximize species distribution and minimizes the proportional loss of species ranges for limited-range species (Moilanen, Possingham & Polasky, 2009). We also calculated the mean number of species represented in 100 sites selected at random—null model (*R*).

SAI is formally defined as SAI = (*S-R*)/(*O-R*). Values can range from −∞ to + ∞. Negative values indicate that the surrogate is worse than a null model, while values that are close 0 are comparable to those obtained by the random solutions. Positive values represent a measure of efficiency by the surrogate. SAI scores were calculated for 201 targets, which represent the percentages of the area of our hypothetical “reserve”. Targets ranged from 10% to 30% at 0.1% intervals. We used the mean of these 201 SAI scores to obtain an estimate of the overall performance of each rarity index. We used the Friedman rank-sum test (Friedman, 1937) to estimate if the solutions calculated by rarity and richness (SAI values) differ among groups. Friedman is a nonparametric test that compares three or more related (i.e., dependent) samples (Friedman, 1937). Also, we used the *posthoc.friedman.nemenyi.test* function in R to calculate the pairwise comparisons using Nemenyi post-hoc test for unreplicated blocked data (Pohlert, 2014).

## Results

**Figure 1:**
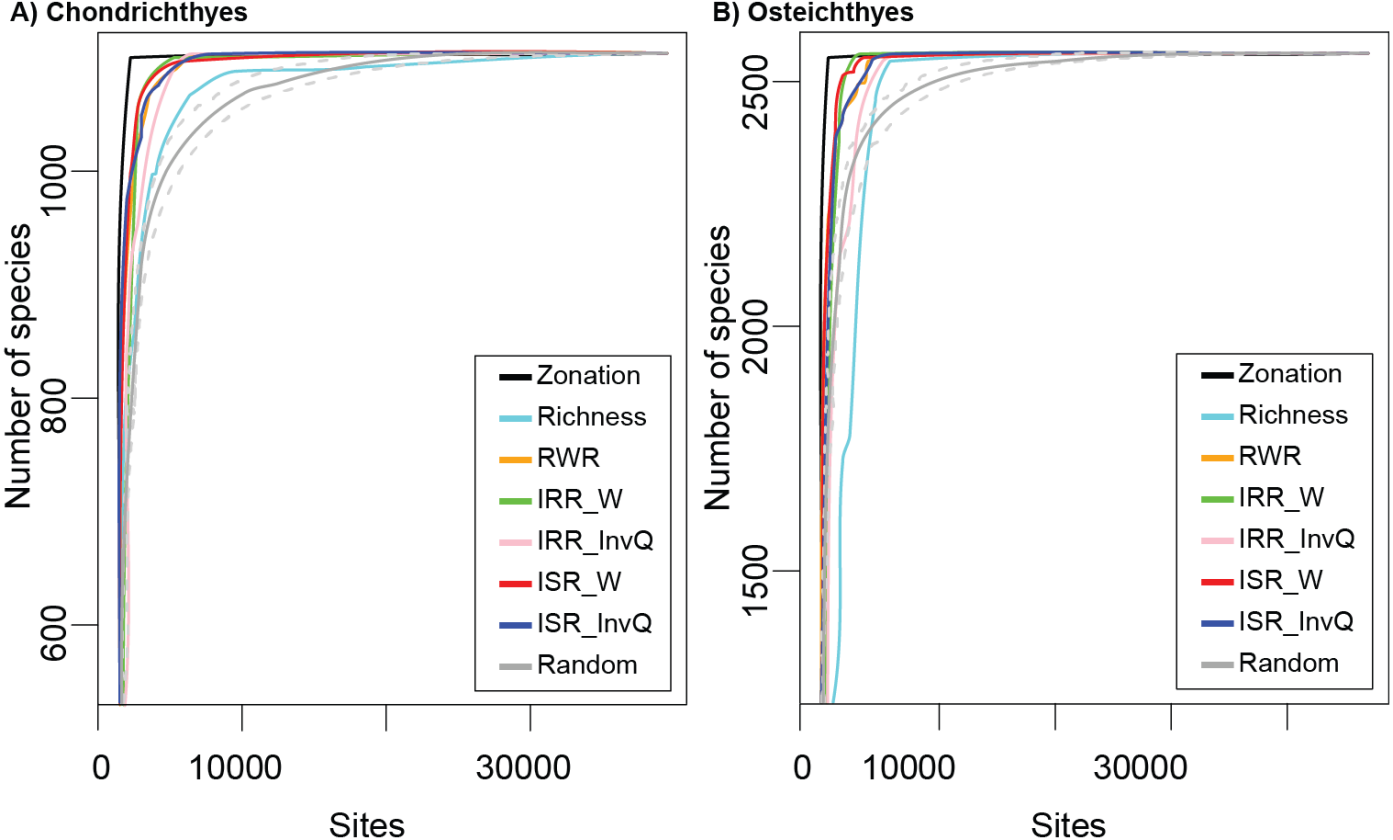
Species accumulation curves for (A) Chondrichthyes and (B) Osteichthyes. Zonation represents the optimum solution as calculated by complementarity and Richness represents the alpha diversity solution. Rarity indices are: RWR, rarity-weighted richness; IRR_W, index of relative rarity calculated using *W*; IRR_InvQ, index of relative rarity calculated using *invQ*; ISR_W, index of summed rarity calculated using *W*; and ISR_InvQ, index of summed rarity calculated using *invQ*. Random solution is represented with the 95% confidence interval.

The cumulative number of species recorded using complementarity, richness, and rarity (RWR, ISR, and IRR) indices were not consistent across all marine fish taxonomic groups tested (Figs. 1A and 1B). Based on species accumulation curves (Figs. 1A and 1B), sites selected by the complementarity solution, represented by Zonation, provided better coverage than the set of selected random cells. The performance of complementarity as an indicator of fish biodiversity, however, was only slightly better than the performance of rarity. Similarly, rarity solutions accumulated more species than the random solution and worked better than richness in all cases. The results of richness varied depending on the group. For the Chondrichthyes (Fig. 1A), richness always provided better coverage than random, while for the Osteichthyes, the cumulative number of species was lower than random in several sites (Fig. 1B).

**Figure 2:**
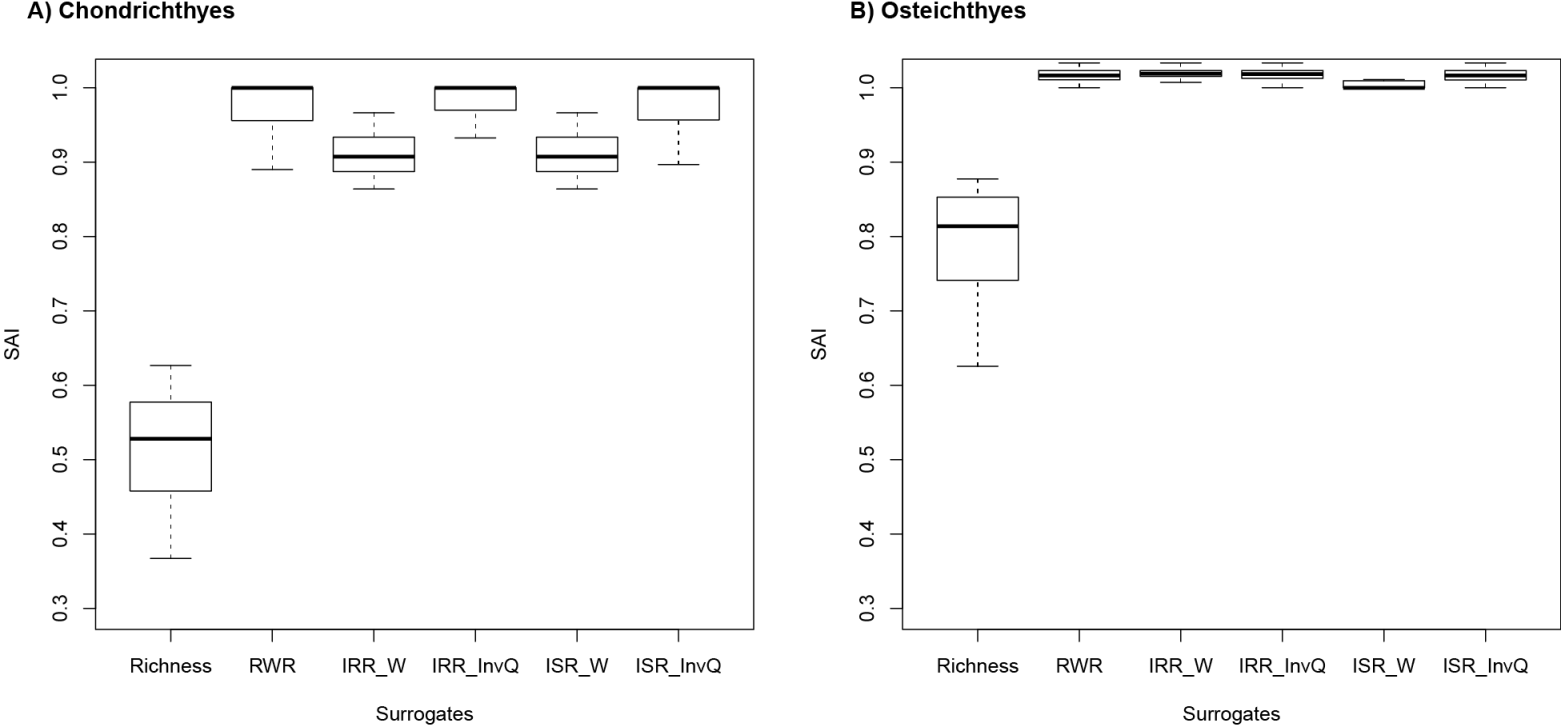
Species accumulation index (SAI) scores for (A) Chondrichthyes and (B) Osteichthyes. Species accumulation index (SAI) scores for 201 targets (0.1–30% by 0.1% increments) for (A) Chondrichthyes and (B) Osteichthyes. Negative values indicate solutions worse than random. Positive values are a measure of effectiveness (e.g., a value of 1.0 means a result is 100% as effective as the optimum solution, as calculated by Zonation). Richness represents the alpha diversity solution. Rarity indices are: RWR, rarity-weighted richness; IRR_W, index of relative rarity calculated using *W*; IRR_InvQ, index of relative rarity calculated using *invQ*; ISR_W, index of summed rarity calculated using *W*; and ISR_InvQ, index of summed rarity calculated using *invQ*.

SAI scores revealed that rarity indices were more effective than species richness in all cases (Fig. 2A and 2B). For Chondrichthyes, SAI values for species richness ranged from 0.35 to 0.51 and from 0.85 to 1.0 for rarity indices. For Osteichthyes, richness scores were higher, with values ranging from 0.43 and 0.78. SAI for rarity indices ranged from 0.86 to 1.033. In several instances, rarity indices had mean efficiency scored at 100%, which indicates that rarity is as good as the complementary approach (the near-optimum solution) at improving on random results (Fig. 1). SAI for rarity values had greater values than complementarity in 73 (36.3%) and 188 (93.6%) instances for Chondrichthyes and Osteichthyes, respectively.

The Friedman tests indicated that SAI values differ among richness and rarity indices—Chondrichthyes Friedman chi-squared = 776.17 and *p* < 0.0001; Osteichthyes–Friedman chi-squared = 745.12 and *p* < 0.0001. Post-hoc tests show that richness SAI values differ from all rarity indices in all cases (Table 1). Differences between rarity indices were observed, but only for a few instances.

**Table 1:** Pairwise comparisons of species accumulation values (SAI), as estimated by richness and rarity solutions, using Nemenyi post-hoc test for dependent data. This test calculates the levels of significance, represented by *p*-values. Bold values indicate significant differences in SAI values. Richness represents the alpha diversity solution.

	Richnes	RWR	IRR_W	IRR_invQ	ISR_W	IRS_invQ
**Indice**	**Chondrichthyes**					
RWR	**<0.001**	–	–	–	–	–
IRR_W	**<0.001**	**<0.001**	–	–	–	–
IRR_invQ	**<0.001**	**0.0013**	**<0.001**	–	–	–
ISR_W	**<0.001**	**<0.001**	1.00	**<0.001**	–	–
IRS_invQ	**<0.001**	0.9998	**<0.001**	**0.0037**	**<0.001**	–
	**Osteichthyes**					
RWR	**<0.001**	–	–	–	–	–
IRR_W	**<0.001**	**0.0097**	–	–	–	–
IRR_invQ	**<0.001**	1.00	**0.0093**	–	–	–
ISR_W	**<0.001**	**<0.001**	**<0.001**	**<0.001**	–	–
IRS_invQ	**<0.001**	1.00	**0.0053**	1.00	**<0.001**	–

**Notes.**

Rarity indices are RWR rarity-weighted richness IRR_W index of relative rarity calculated using *W* IRR_InvQ index of relative rarity calculated using *invQ* ISR_W index of summed rarity calculated using *W* ISR_InvQ index of summed rarity calculated using *invQ*

## Discussion

The performance of rarity indices has been previously investigated in terrestrial systems (Csuti, 1997; Albuquerque & Beier, 2015c; Astudillo-Scalia & Albuquerque, 2019); however, to the best of our knowledge, this is the first time that rarity indices, expressed by RWR, ISR, and IRR, are assessed as surrogates of fish biodiversity in global marine systems. Our results support the hypothesis that rarity is a reliable solution for representing most species in a small number of sites requiring at least one occurrence of each species at marine realms and especially for fish diversity. Results also show that SAI values were consistent across all fish groups tested. Our results are significant because it opens a new area of collaboration between biogeography and marine conservation biology since planners can use biogeographical patterns of rarity to enhance the performance of the current protected area network. If most high-rarity sites are not protected, planners could determine if species in the unprotected rarity hotspots lack additional protection.

While we acknowledge that rarity indices cannot replace optimal solutions for minimizing biological loss, our results suggest that rarity can be useful for estimating near-optimum solutions. Near-optimal solutions identify areas that effectively achieve conservation objectives and represent a benchmark in spatial conservation prioritization planning (Moilanen, Possingham & Polasky, 2009). Our findings show that rarity (calculated using RWR, *W* and *invQ* method) was extremely effective at producing results within the targets for the marine fish groups tested (Table 1), a result that is consistent with previous surrogacy analyses in terrestrial ecosystems (Albuquerque & Beier, 2015a; Albuquerque & Beier, 2015b; Albuquerque & Beier, 2015c; Albuquerque & Beier, 2016; Astudillo-Scalia & Albuquerque, 2019). Rarity indices show a 100% efficiency rate, according to SAI scores (Fig. 2). This high efficiency is, partially explained by the way rarity indices score sites, since areas with rare species receive higher conservation values than sites with common species (Ratcliffe, 1977). For example, a site with a unique rare species would have high conservation value, and therefore it would be selected by the conservation prioritization solutions at the early stages of the accumulation process (Fig. 1). Otherwise, if the same weight is uniformly applied among species, the cell with only one rare species would receive a lower score and would be included in the late stage of prioritization. In such cases, rare species are not likely to be included in sites selected to protect species. Another advantage is computing time. Rarity indices can be obtained in a few seconds (real-time decision). When time is critical, other approaches that incur longer waiting times might delay the process of conservation planning (Pressey, Possingham & Margules, 1996). Rarity metrics also do not require extensive analytic or programming skills and specialized geographical software. Results can be produced by using a spreadsheet or open-source software (e.g., R; R Core Team, 2019).

Rarity results were comparable to or better than those obtained by the complementarity approach, in several instances (Fig. 2). This does not mean that both solutions prioritize the same cells. Rarity utilizes the weight assigned to species given their level of rarity to prioritize species with limited ranges over widespread species (Leroy et al., 2012; Leroy, Canard & Ysnel, 2013). Complementarity algorithms, on the other hand, start by selecting sites with the highest value to minimize biological loss. The subsequent site selection is assembled with sites that add new species (as opposed to continuing to select sites with a high number of species) (Pressey, Possingham & Margules, 1996; Moilanen et al., 2014).

Similar to that reported by previous studies in terrestrial realms (Albuquerque & Beier, 2015c; Veach et al., 2017), we found that species richness was the least effective surrogate in terms of efficiently covering Chondrichthyes and Osteichthyes diversity (Fig. 2 and Table 1). Because richness fails to represent the most number of species in the smallest number of sites (Kirkpatrick, 1983), the use of richness as a surrogate of species representation in this context is often inadequate. The low efficiency found herein may be related to the way richness assembles the solutions. Sites with the highest number of species often have greater conservation values, irrespective of the status of the species present in those sites (Astudillo-Scalia & Albuquerque, 2019). As a consequence, richness solutions often assemble sites that share the same species composition and leave sites with a low number of rare species out (Veach et al., 2017).

Our results are planned for hypothetical tests of the effectiveness of rarity as a surrogate of marine fish biodiversity. First, although range maps represent the geographic distribution of species, they do not necessarily describe their actual area of distribution, since range maps tend to overestimate the presence of species (Di Marco et al., 2017). Also, we acknowledge that results found herein could be masked by the geographical extent of this study since priority areas not always are designed at the same resolution of our study. Similarly, the spatial scale of each solution may not be in accordance with the scale at which richness and rarity are measured. Second, our study lacks specific information such as life-history (e.g., breeding and feeding), connectivity between priority areas, the financial costs associated with the management and expansion of a reserve or protected area (Pressey, Possingham & Margules, 1996). Yet, we believe that our results justify further tests to evaluate whether sites selected by rarity indices will efficiently represent species, especially at finer scales.

## Conclusion

Overall, our study confirms that rarity is a valid surrogate of fish diversity. Results also show rarity indices were as effective as the complementarity-based solution (optimal solutions), in most of the cases. Ours is the first assessment of the performance of RWR, ISR, and IRR in marine fish species at a global scale, and in this context, we believe the use of rarity indices as surrogates for conservation in site prioritization of marine fishes could be a promising approach. Increasing our knowledge and use of conservation tools can significantly increase our ability to counteract the current biodiversity loss crisis.

##  Supplemental Information

10.7717/peerj.8373/supp-1 Supplemental Information 1 Raw dataset depicting the presence and absence of fishes at a global scale Each data set represents the matrix of presence and absence of fish species at a global scale. The species richness, complementarity values (as expressed by Zonation) and rarity values are also included.

## Additional Information and Declarations

### Data Availability

The following information was supplied regarding data availability: The raw measurements are available in the Supplementary Files.
